# Prospective Evaluation of Fractional Carbon Dioxide Laser Treatment of Mature Burn Scars, Post-traumatic Scars, and Post-acne Scars

**DOI:** 10.7759/cureus.58358

**Published:** 2024-04-16

**Authors:** Yasharth Sharma, Pradeep Jain, Suman Babu Gottam, Arnab Sarkar, Nikhil Prasad

**Affiliations:** 1 Plastic and Reconstructive Surgery, Moti Lal Nehru Medical College, Prayagraj, IND; 2 Plastic and Reconstructive Surgery, Banaras Hindu University, Varanasi, IND

**Keywords:** posas scale, fractional carbon dioxide laser, acne scar, burn scar, traumatic scar

## Abstract

Background: Annually, around 100 million patients worldwide acquire scars, some of which can cause significant problems. Various treatment interventions, such as topical scar creams, steroids, laser therapy, and surgery, have been developed to manage these scars. This study was conducted to evaluate the effectiveness of fractional CO_2_ laser treatment by assessing outcomes using the Patient Observer Scar Assessment Scale (POSAS) and clinical photographs.

Materials and methods: A total of 47 patients were included in the study, divided into three groups: a post-acne scar group with 14 patients, a post-burn scar group with 17 patients, and a post-traumatic scar group with 16 patients. Detailed histories were taken, and clinical examinations were performed and recorded on a prepared proforma. Aesthetic outcomes were evaluated based on clinical photographs, and total patient and observer scores were recorded using POSAS at baseline, and after one and three months. POSAS comprises two components: the observer scale (POSAS-O) and the patient scale (POSAS-P). Fractional CO_2_ laser treatments were performed in each group, with sessions repeated every four weeks for three consecutive sessions. Data were analyzed using the paired t-test for before-and-after comparisons in each study group. Welch's ANOVA test was used for comparisons among the three groups at a significance level of p=0.05, using MS Excel (Microsoft Corporation, Redmond, Washington) and IBM SPSS Statistics for Windows, Version 20 (Released 2011; IBM Corp., Armonk, New York).

Results: The mean age for men was 26.38 ± 8.19 years and for women 22.21 ± 6.38 years. The study comprised 34 female patients (72.34%) and 13 male patients (27.66%). The mean POSAS observer and patient scales were recorded and compared for all three types of scars from baseline to three months. The mean percentage change in POSAS-O and POSAS-P (total score) in relation to different scar sites was recorded. The most significant difference in mean percentage change, statistically significant (p-value < 0.05), was observed for facial scars, followed by scars on the neck, and was minimal for scars on the hand, in both observer and patient groups. Even a single session of fractional CO_2_ laser therapy had profound effects on the overall quality of scars.

Conclusion: Fractional carbon dioxide laser therapy improves the quality of scars and produces significant improvements in skin texture, with better effects on post-traumatic scars than on post-burn and post-acne scars. Future studies are needed to better understand the mechanism of action and to optimize the doses and timing of therapy.

## Introduction

Around 100 million patients acquire scars worldwide annually, of which approximately 11 million are keloid and four million are burn scars, 70% of which occur in children. Abnormal skin scarring can cause psychological, aesthetic, social, and physical consequences [1,2]. Worldwide, millions of individuals develop traumatic scars each year due to burns and other traumatic injuries [2]. Scars commonly form at the site of tissue injury and can be of atrophic or hypertrophic types, with hypertrophic scars showing a prevalence rate of 70% [3].

Acne is another common skin ailment with a prevalence rate of around 90% in adolescents and 12%-14% among adults, causing significant psychological and social implications [4]. Acne scars are broadly of two types-atrophic scars and hypertrophic scars or keloids. Atrophic scars are almost three times more common than hypertrophic scars and keloids and are further categorized into icepick scars (60-70%), boxcar scars (20-30%), and rolling scars (15-25%) [4]. The severity of permanent scars from inflammatory acne lesions depends on delays in managing acne patients. It has been observed that pathological scars form due to dysregulation in the wound healing process, which includes an inflammatory, proliferative phase, and a remodeling phase [5-6]. Pain, pruritus, and major morbidities such as deformities of facial architecture and a restricted range of motion in functional joints are common signs and symptoms [7].

Several treatment interventions have been developed for managing traumatic scars. These treatment options are divided into surgical and non-surgical approaches [8]. Currently, surgical treatment is one of the mainstream approaches for treating traumatic scars, including post-burn scars. Surgical intervention in acne scars is primarily in the form of subcision. However, being an invasive method, surgery has an increased risk of inducing the formation of new scars and can lead to the recurrence of scars. Whereas, the non-surgical approaches for treating traumatic scars consist of massage therapy, application of silicone gel, compression garments, radiation therapy, intralesional therapy using steroids and other medications (like 5-FU, bleomycin, mitomycin C, TGF-beta 3, and interferon), botulinum toxin, laser, and light therapy [9]. These new techniques are supposed to have various advantages of being minimally invasive, showing a faster recovery rate, and having a low risk of adverse effects.

Thus, treatment of scars is challenging and various therapeutic options are available for the treating plastic surgeon. Among the different treatment options, the literature reports the successful use of lasers, both ablative and nonablative devices, for managing atrophic and hypertrophic burn scars, acne scars, and traumatic scars, with or without the use of PRP (platelet-rich plasma) as an alternative to surgical correction [9].

Therefore, the present study was conducted to evaluate the effectiveness of fractional CO_2_ laser treatment on mature burn, post-traumatic, and post-acne scars by assessing the aesthetic outcomes. This assessment considered patient symptoms such as pain, itching, color, and stiffness, as well as scar characteristics including vascularity, pigmentation, pliability, and thickness. These evaluations were made using the Patient Observer Scar Assessment Scale (POSAS) and clinical photographs.

## Materials and methods

This prospective observational study was conducted at the Institute of Medical Sciences (IMS), Banaras Hindu University (BHU) from September 2019 to December 2021, after receiving ethical clearance from the institutional ethics committee (IEC No. Dean/2020/EC/1905). This work was carried out in accordance with the Code of Ethics of the World Medical Association (Declaration of Helsinki) for experiments involving humans. Informed consent was obtained from all patients. A total of 90 patients with mature post-acne, post-burn, and post-traumatic scars were initially included in the study, with 30 patients in each category. However, patients with unstable scars, hypertrophic scars, associated malignancies, and contractures were excluded from the study. Additionally, patients who had received any treatment for scars in the previous three months were also excluded.

However, due to the COVID-19 pandemic, the sample size in all three groups (n=47) fell short of the proposed size. Consequently, the final groups comprised 14 patients in the post-acne scar group, 17 in the post-burn scar group, and 16 in the post-traumatic scar group. The procedure was explained in detail, and duly signed informed consent was obtained from each patient before the procedure was carried out.

A detailed history was taken and a clinical examination was performed and recorded for each patient on a prepared proforma. Photographs were taken at every visit and recorded in digital format. Preoperative POSAS observer scale (POSAS-O) scoring was conducted by the principal investigator, and the patient scale (POSAS-P) scoring was conducted by the patients themselves under the supervision of the principal investigator (POSAS stands for Patient and Observer Scar Assessment Scale).

The observer scale of the Patient and Observer Scar Assessment Scale (POSAS) consists of six items: vascularity, pigmentation, thickness, relief, pliability, and surface area. Each item is scored on a scale that ranges from 1, denoting 'like normal skin,' to 10, indicating the 'worst scar imaginable.' The sum of these six items constitutes the total score of the POSAS observer scale. Additionally, an overall opinion is also scored on a scale from 1 to 10. Vascularity is assessed by observing the presence of vessels in the scar tissue, which is tested by the amount of blood return after blanching with a piece of Plexiglas. Pigmentation refers to the brownish coloration of the scar caused by pigment (melanin). Thickness is measured as the average distance between the subcutical-dermal border and the epidermal surface of the scar. Relief measures the extent to which surface irregularities are present. Pliability denotes the suppleness of the scar, and surface area is gauged in relation to the original wound area.

Similarly, the patient scale of the POSAS includes six questions that are each scored on a scale from 1 to 10. These questions address whether the scar has been painful or itchy in the past few weeks, whether the scar color, stiffness, thickness, or irregularity differs from that of normal skin. The cumulative score of these items results in the total score of the POSAS patient scale, with an additional overall opinion being scored on a scale from 1 to 10.

The aesthetic outcome was evaluated based on clinical photographs, and the total patient and observer scores were recorded using the POSAS scale after three months. We made a slight modification to the POSAS scale to maintain uniformity among the three groups. In the POSAS-O scale, the parameters of thickness and surface area were consistently assigned a score of 1, reflecting our study’s exclusion of hypertrophic scars. Similarly, in the POSAS-P group, the thickness parameter was assigned a score of 1 throughout. For comparative analysis among the three groups, the percentage change in total scores (µΔTS) was calculated. Pain was scored on a numerical rating scale from 0 to 10, with 0 representing no pain and 10 representing the worst pain imaginable.

Fractional CO_2_ laser treatments were performed using the SmaXel CO_2_ laser (IDS Ltd., Republic of Korea) with pre-installed settings: energy/dot of 45 millijoules, pulse duration of 1.9 milliseconds, density level of 15, and depth level of 1 for post-traumatic and post-burn scars. For post-acne scars, the settings were energy/dot of 42 millijoules, pulse duration of 1.8 milliseconds, density level of 12, and depth level of 1. Epidermal cooling with ice rollers was applied for 15 minutes before each session. Post-treatment, the area was covered with a thin layer of mupirocin ointment. For pain control, patients were advised to take NSAIDs as needed. Treatments were repeated every four weeks for three consecutive sessions. Twelve weeks after the last treatment, patients completed the POSAS again. Digital photographs were taken at the initial (before treatment) and follow-up (after treatment) visits. The data collected were statistically analyzed using the t-test for before-and-after comparisons in each study group and Welch’s ANOVA test for comparisons among the three groups, at a significance level of p=0.05, using MS Excel (Microsoft Corporation, Redmond, Washington) and IBM SPSS Statistics for Windows, Version 20 (Released 2011; IBM Corp., Armonk, New York).

## Results

The most common age group in all three study groups was 21-25 years, with the mean age for men and women being 26.38 ± 8.19 and 22.21 ± 6.38 years, respectively. The female-to-male sex ratio in the post-traumatic, post-burn, and post-acne scar groups was 1.7:1, 7.5:1, and 1.8:1, respectively, with total females and males being 34 (72.34%) and 13 (27.66%). The mean duration of scars was 6.30 ± 6.93 years, ranging from 2 tp 16 years. The most common site of scars was the face (68.08%), followed by the hands (10.64%) and abdomen (8.51%). In the post-acne, post-traumatic, and post-burn scar groups, the face was involved 100%, 50%, and 59% of the time, respectively.

In the post-traumatic scar observer assessment group, all variable parameters except pigmentation showed statistically significant (p-value <0.05) improvement after one month. Similarly, in the patient assessment group, all variable parameters demonstrated statistically significant improvement. The parameters of pain and itching also improved, although the differences were not statistically significant (p-value > 0.05).

In the post-burn scar observer assessment group, all variable parameters except pigmentation and relief showed statistically significant improvement. In the patient assessment group, all variable parameters showed improvement, but the difference was statistically significant only in the case of stiffness of the scar, whereas pain and itching showed no change.

**Figure 1:**
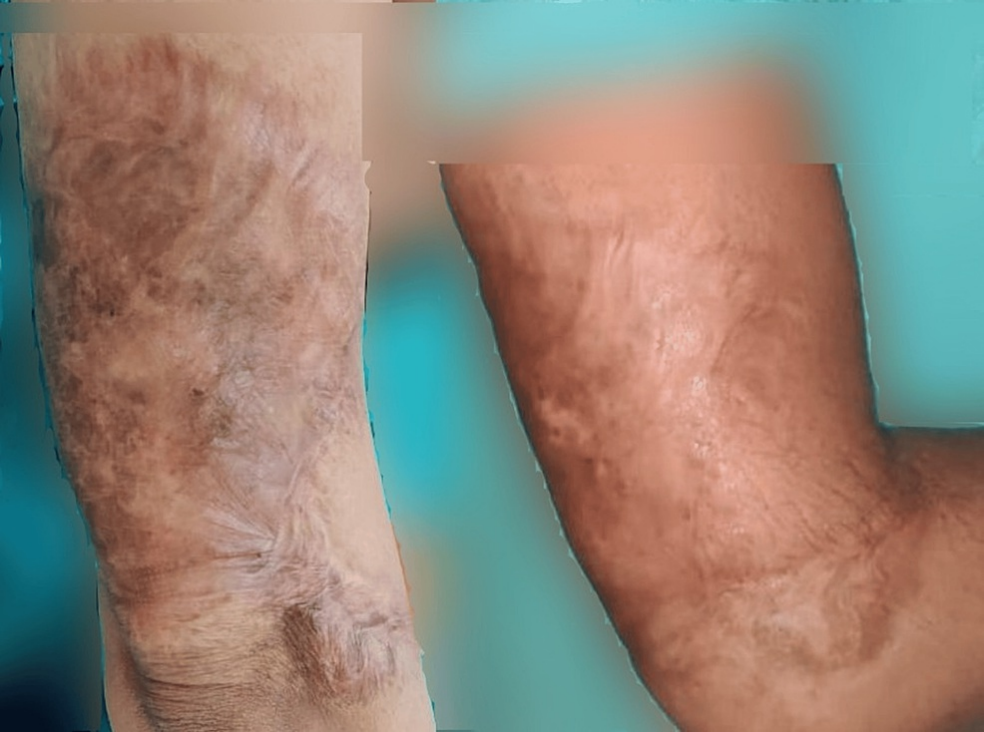
Pre- and post-laser therapy results in a burn scar patient at three months after last session The picture on the left serves as a baseline reference, taken before the initial laser treatment. The picture on the right was captured at the three-month follow-up, after the completion of the last laser session. Site of scar: Lateral aspect of the right arm and elbow.

**Figure 2:**
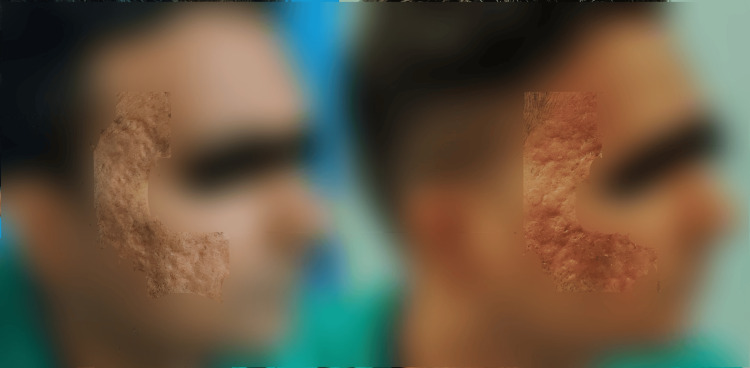
Pre- and post-laser therapy results in a patient with acne scar at three months after last session The picture on the left serves as a baseline reference, taken before the initial laser treatment. The picture on the right was captured three months after the last laser session, showing the treatment outcome. Site of scar: Right side of the temple and cheek.

**Figure 3:**
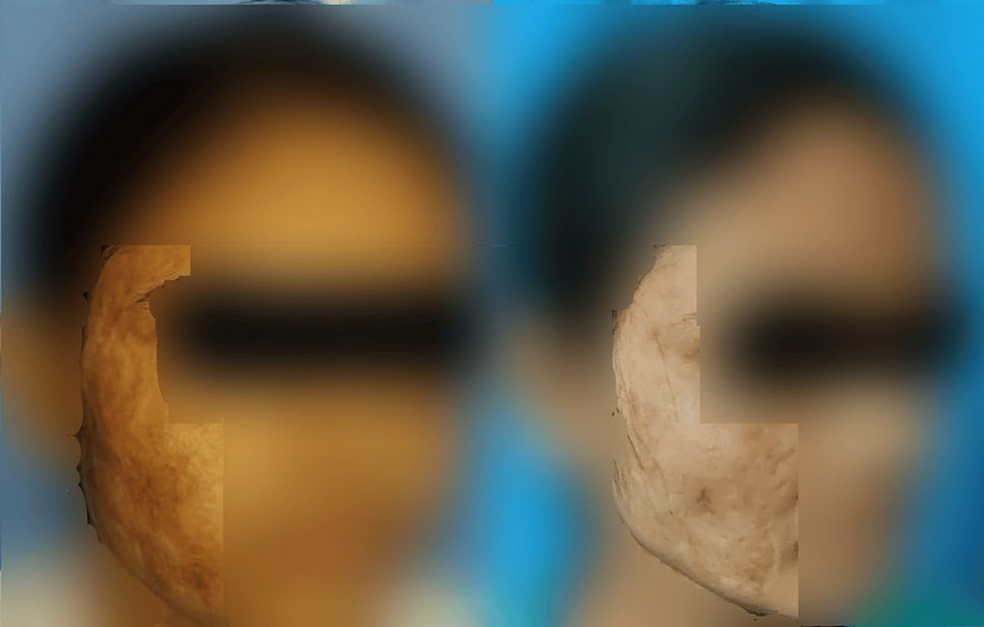
Pre- and post-laser therapy results in a patient with traumatic scar at three months after the last session The picture on the left is a baseline reference taken before the initial laser treatment, while the picture on the right was captured three months after the last laser session, showing the treatment outcome. Site of scar: Right side of temple and cheek.

In the post-acne scar observer assessment group, statistically significant improvements were observed in relief, pliability, and total score. Similarly, in the post-acne patient assessment group, statistically significant improvements were found for color, stiffness, irregularity, overall opinion, and total score. Results for three patients (one from each group) are shown in Figures 1-3.

The mean POSAS observer and patient scales were recorded and compared for all three types of scars from baseline to three months, as detailed in Tables 1-6. The mean percentage change in POSAS-O and POSAS-P (total score) in relation to different scar sites is documented in Table 7. The demographic profiles of the patients in the three groups are presented in Tables 8-10.

**Table 1:** Mean POSAS observer scale (trauma) *Difference is statistically significant (p<0.05) ^!^Excluded from the scoring All items are scored on a scale ranging from 1 (like normal skin) to 10 (worst scar imaginable) The sum of the six items results in a total score on the POSAS observer scale Overall opinion is scored on a scale ranging from 1 to 10 A lower score is better than a higher score as depicted in the Appendix The range of the total score is 6-60. The lower the score, the better it is.

Parameters	Baseline	One month after the first session	Three months after the third session
Vascularity^*^	4.62 ± 2.72	4.13 ± 1.02	2.38 ± 1.91
Pigmentation*	3.31 ± 3.70	3.19 ± 1.72	2.12 ± 1.91
Thickness^!^	1 ± 0	1 ± 0	1 ± 0
Relief^*^	4.75 ± 3.39	4.38 ± 1.41	2.31 ± 2.50
Pliability^*^	4.56 ± 3.42	3.81 ± 1.28	2.38 ± 2.05
Surface area^!^	1 ± 0	1 ± 0	1 ± 0
Overall opinion^*^	5.25 ± 2.25	4.75 ± 0.77	2.75 ± 1.55
Total score^*^	19.25 ± 6.71	17.5 ± 2.63	11.19 ± 6.12

**Table 2:** Mean POSAS patient scale (trauma) *Difference is statistically significant (p<0.05) **Difference is statistically insignificant (p>0.05) ^!^Excluded from the scoring All items are scored on a scale ranging from 1  to 10  according to the questionnaire in the appendix The sum of the six items results in a total score on the POSAS patient scale Overall opinion is scored on a scale ranging from 1 to 10 A lower score is better than a higher score as depicted in the Appendix The range of the total score is 6-60. The lower the score, the better it is.

Parameters	Baseline	One month after the first session	Three months after the third session
Painful scar^**^	1.12 ± 0.34	1 ± 0	1 ± 0
Scar itching**	1.25 ± 0	1.06 ± 0.25	1 ± 0
Scar color different from normal skin ^*^	5.69 ± 1.40	5.44 ± 1.46	3.31 ± 1.20
Stiffness of scar different from normal skin*	5.62 ± 3.17	4.94 ± 1.34	3.44 ± 1.93
Thickness of scar different from normal skin^!^	1 ± 0	1 ± 0	1 ± 0
Irregular scar^*^	4.88 ± 1.26	4.63 ± 1.15	2.88 ± 0.72
Overall opinion^*^	5.69 ± 0.95	5.63 ± 0.89	3.56 ± 0.81
Total score^*^	19.56 ± 2.76	18.25 ± 2.46	12.63 ± 2.19

**Table 3:** Mean POSAS observer scale (burn) *Difference is statistically significant (p<0.05) ^!^Excluded from the scoring All items are scored on a scale ranging from 1 (like normal skin) to 10 (worst scar imaginable) The sum of the six items results in a total score on the POSAS observer scale Overall opinion is scored on a scale ranging from 1 to 10 A lower score is better than a higher score as depicted in the Appendix The range of the total score is 6-60. The lower the score the better it is.

Parameters	Baseline	One month after the first session	Three months after the third session
Vascularity^*^	5.12 ± 1.17	4.88 ± 0.99	3.12 ± 1.05
Pigmentation*	5.06 ± 1.89	4.94 ± 1.82	3.12 ± 1.45
Thickness^!^	1 ± 0	1 ± 0	1 ± 0
Relief^*^	4.24 ± 1.75	4.06 ± 1.60	2.47 ± 1.01
Pliability^*^	4.29 ± 1.93	3.65 ± 1.45	2.65 ± 1.17
Surface area^!^	1 ± 0	1 ± 0	1 ± 0
Overall opinion^*^	5.59 ± 0.93	5.29 ± 0.92	3.41 ± 0.87
Total score^*^	20.71 ± 3.48	19.47 ± 3.16	13.35 ± 3.02

**Table 4:** Mean POSAS patient scale (burn) *Difference is statistically significant (p<0.05) ^!^Excluded from the scoring ^£^No change All items are scored on a scale ranging from 1 to 10 according to the questionnaire in the Appendix The sum of the six items results in a total score on the POSAS patient scale Overall opinion is scored on a scale ranging from 1 to 10 A lower score is better than a higher score as depicted in the Appendix The range of the total score is 6-60. The lower the score, the better it is.

Parameters	Baseline	One month after the first session	Three months after the third session
Painful scar^£^	1 ± 0	1 ± 0	1 ± 0
Scar itching^£^	1 ± 0	1 ± 0	1 ± 0
Scar color different from normal skin ^*^	4.76 ± 2.20	4.59 ± 2.06	2.76 ± 1.03
Stiffness of scar different from normal skin^*^	5.18 ± 1.07	4.53 ± 0.94	3.06 ± 0.83
Thickness of scar different from normal skin^!^	1 ± 0	1 ± 0	1 ± 0
Irregular scar^*^	3.18 ± 1.24	3 ± 1	2.12 ± 0.70
Overall opinion^*^	5.88 ± 1.22	5.65 ± 1.06	3.24 ± 0.90
Total score^*^	16.12 ± 3.24	15.12 ± 2.73	10.94 ± 1.82

**Table 5:** Mean POSAS observer scale (acne) *Difference is statistically significant (p<0.05) ^!^Excluded from the scoring All items are scored on a scale ranging from 1 (like normal skin) to 10 (worst scar imaginable) The sum of the six items results in a total score on the POSAS observer scale Overall opinion is scored on a scale ranging from 1 to 10 A lower score is better than a higher score as depicted in the Appendix The range of the total score is 6-60. The lower the score, the better it is.

Parameters	Baseline	One month after the first session	Three months after the third session
Vascularity^*^	2.14 ± 0.95	1.93 ± 0.83	1.36 ± 0.50
Pigmentation^*^	4.21 ± 1.67	4.08 ± 1.5	2.64 ± 1.55
Thickness^!^	1 ± 0	1 ± 0	1 ± 0
Relief^*^	4.00 ± 1.57	3.43 ± 1.22	2.36 ± 0.84
Pliability^*^	3.57 ± 1.65	3 ± 1.18	1.86 ± 1.10
Surface area^!^	1 ± 0	1 ± 0	1 ± 0
Overall opinion^*^	4.71 ± 1.20	4.43 ± 1.22	2.79 ± 0.80
Total score^*^	15.93 ± 2.92	14.36 ± 2.37	10.21 ± 1.97

**Table 6:** Mean POSAS patient scale(acne) *Difference is statistically significant (p<0.05) **Difference is statistically not significant (p>0.05) ^!^Excluded from the scoring All items are scored on a scale ranging from 1  to 10 according to the questionnaire in the Appendix The sum of the six items results in a total score on the POSAS patient scale Overall opinion is scored on a scale ranging from 1 to 10 A lower score is better than a higher score as depicted in the Appendix The range of the total score is 6-60. The lower the score, the better it is.

Parameters	Baseline	One month after the first session	Three months after the third session
Painful scar^**^	1.21 ± 0.58	1.07 ± 0.27	1 ± 0
Scar itching^**^	1.29 ± 0.61	1.07 ± 0.27	1 ± 0
Scar color different from normal skin ^*^	5.50 ± 1.22	4.79 ± 1.19	3.50 ± 0.85
Stiffness of scar different from normal skin^*^	5.36 ± 1.55	4.14 ± 1.01	3.21 ± 0.89
Thickness of scar different from normal skin^!^	1 ± 0	1 ± 0	1 ± 0
Irregular scar ^*^	5.29 ± 1.33	4.36 ± 0.84	2.93 ± 0.73
Overall opinion^*^	5.43 ± 1.45	5.07 ± 1.21	3.36 ± 0.74
Total score^*^	19.64 ± 2.79	16.5 ± 2.31	12.64 ± 1.34

**Table 7:** Mean percentage change in POSAS-O and POSAS-P (total score) in relation to different scar sites ^#^Significant difference in both POSAS-O as well as POSAS-P group (p-value <0.05) ^$^No significant difference in both groups ^Ω^Indeterminate µΔTS%: Mean of percentage change in total score.

Site	POSAS-O (µΔTS%)			POSAS-P (µΔTS%)		
	Trauma	Burn	Acne	Trauma	Burn	Acne
Face^#^	52.69 ± 10.31	41.02 ± 11.82	35.59 ± 14.89	43.08 ± 5.70	36.59 ± 13.23	35.27 ± 8.30
Arm^$^	35.83 ± 30.64	27.92 ± 1.84	_	30.92 ± 15.75	26.05 ± 7.13	_
Abdomen^$^	26.90 ± 8.75	25.40 ± 8.98	_	29.29 ± 2.02	21.76 ± 4.99	_
Hand^$^	23.17 ± 13.14	28.83 ± 7.77	_	20.40 ± 12.90	19.05 ± 6.73	_
Neck^Ω^	47.62	33.33	_	36.84	30.77	_

**Table 8:** Demographic profile of post-traumatic scar patients

S. No.	Age (years)	Sex	Scar site	Duration of scar (years)	Previous treatment
T1	18	F	Face	7	-
T2	25	F	Hand	2	-
T3	20	F	Face	8	Topical
T4	26	M	Face	7	Topical
T5	24	F	Hand	6	Topical
T6	30	M	Neck	4	-
T7	21	F	Face	7	Topical
T8	26	M	Abdomen	3	-
T9	28	F	Arm	9	-
T10	21	M	Face	3	Topical
T11	30	M	Abdomen	5	-
T12	22	F	Face	3	Topical
T13	27	F	Face	6	Topical
T14	18	F	Arm	5	-
T15	23	F	Hand	4	-
T16	24	M	Face	2	

**Table 9:** Demographic profile of post-burn scar patients

S. No.	Age (years)	Sex	Scar site	Duration of scar (years)	Previous treatment
B1	20	F	Face	3	Topical
B2	17	F	Face	16	Topical
B3	23	F	Abdomen	4	None
B4	22	F	Arm	5	Topical
B5	33	M	Hand	11	None
B6	24	F	Face	5	Surgery
B7	26	F	Abdomen	3	Topical
B8	29	M	Face	7	None
B9	18	F	Face	2	None
B10	27	F	Neck	4	None
B11	22	F	Arm	3	None
B12	28	F	Face	8	Topical
B13	19	F	Face	3	Topical
B14	23	F	Face	13	Topical
B15	17	F	Hand	2	None
B16	23	F	Face	4	Topical
B17	21	F	Face	3	None

**Table 10:** Demographic profile of post-acne scar patients

S. No.	Age (years)	Sex	Scar site	Duration of scar (years)	Previous treatment
A1	18	F	Face	5	Topical
A2	21	M	Face	7	Topical
A3	24	F	Face	10	Topical
A4	21	F	Face	6	Topical
A5	27	F	Face	12	Topical
A6	21	M	Face	6	Topical
A7	23	F	Face	8	Topical
A8	24	F	Face	8	Topical
A9	19	F	Face	5	Topical
A10	23	M	Face	8	Topical
A11	22	F	Face	7	Topical
A12	30	M	Face	15	Topical
A13	21	F	Face	6	Topical
A14	29	M	Face	13	Topical

The difference in mean percentage change was most pronounced and statistically significant (p-value < 0.05) for facial scars, followed by scars on the neck, and was minimal for scars on the hand, observed in both observer and patient groups.

When comparing the change in total scores relative to the duration of scars, it was found that irrespective of the etiology of the scars, there was no statistically significant difference in either the observer or patient scores for scars older than five years. For scars less than five years old, there was a statistically significant difference (p-value = 0.0222) in patient scores only.

The mean pain score was recorded immediately after the laser treatment and 24 hours later. The pain score was significantly reduced 24 hours post-treatment compared to immediately after the session.

The most common complications, aside from pain, were erythema, followed by crusting and edema, observed in all three groups (52.85%, 26.94%, and 10.88%, respectively). There was no significant difference among the three groups concerning the occurrence of complications. Three instances of bleeding, two of hypopigmentation, and one of post-inflammatory hyperpigmentation (PIH) were observed.

Among the study subjects, 61.7% had undergone topical treatment before enrolling in laser therapy. In the post-burn scar group, one patient had undergone an incomplete scar excision surgery prior to enrollment, having been advised for serial excision but did not return to the operating surgeon for subsequent procedures. In the acne group, all patients had received prior topical therapy. Overall, 63.83% of all patients had already received some form of treatment before enrolling in the study.

## Discussion

This prospective clinical study included patients from diverse backgrounds, skin types, and scar types. Due to the ongoing COVID-19 pandemic, the sample size was reduced from 90 to 47 patients (16 in the post-traumatic group, 17 in the post-burn group, and 14 in the post-acne group). Despite the reduction, the sample size was still two to three times larger than those in similar studies conducted by Salles et al. and Lee et al. [10-11]. Salles et al. included 20 patients with only facial burns, while Lee et al. focused on 14 patients with only post-acne scars.

The mean age of the patients was 26.38 years for men and 22.21 years for women, comparable to the study by Keen et al. [12], where the mean age was 23.2 years. The younger age group in our study may be more concerned about aesthetics and is also more prone to trauma and burns, which explains the age distribution.

The female-to-male ratio in our study was 2.6:1, with 72.34% females and 27.66% males. In contrast, the study by Majid et al. [13] reported a ratio of 1.3:1, with 56% female and 44% male patients, focusing only on non-hypertrophic post-traumatic and post-burn scar patients and a sample size of 25. Our study's higher female-to-male ratio, double that of Majid et al.’s study, likely results from the inclusion of post-acne scar patients and the lack of randomization. However, both studies indicate that more female patients seek laser scar treatment than males.

The duration of scars among our study participants ranged from 2 to 16 years, with a mean of 6.3 ± 6.93 years. For comparison, the study by El-Zawahry et al. [14], which only included 15 patients with post-thermal burn hypertrophic scars or keloids, reported scar durations ranging from 6 months to 30 years.

In our study, 63.83% (30 out of 47) of patients had already undergone some form of treatment before enrolling, similar to the findings of Poetschke et al. [15], where 60% of patients had received some form of therapy. However, in their study, the sample size was only 10 patients, all of whom had post-burn scars only. Since the majority of patients in both studies had undergone some form of treatment before enrolling for laser therapy, it appears that patients may have a lesser tendency to opt for laser scar removal than other forms of treatment. The high cost of laser therapy and the requirement for multiple sessions could be factors influencing this orientation.

In our study, the most common site of scars was the face (68.08%), followed by the hands (10.64%) and the abdomen (8.51%), similar to the sites of scars observed by Godara et al. [16] in their study. Their study involved 67 patients with post-traumatic and post-burn scars, randomly divided into two groups: one treated with fractional CO_2_ therapy and the other with laser therapy combined with PRP. In both groups, the most common site of scars was the face only. Their study noted no difference in the outcome between the groups.

A statistically significant difference was observed in the quality of scars when the baseline individual parameters and total scores of the observer part of POSAS were compared with the scores at the end of the study, across all three groups in question. Such findings have previously been seen in studies by Godara et al. and Blome-Eberwein et al. [16,17]. Blome-Eberwein et al. conducted a prospective study on mature burn scars in 36 patients and found improvements in thickness, sensation, erythema, pigmentation, and elasticity of scars post-fractional CO_2_ therapy. Godara et al. also reported improvements in baseline scar parameters as per POSAS.

Patients in all three groups observed stiffness of scars to respond earliest to the treatment, similar to findings by Godara et al. [16].

Pliability and vascularity were the earliest signs to respond to treatment in our study, while pigmentation was the last. Studies by Keen et al. [12], El-Zawahry et al. [14], and Godara et al. [16] corroborate our findings. It has been observed that fractional carbon dioxide laser (FCL) shows benefits even after one session of laser therapy, as seen in the study by Poetschke et al. [15]. Similarly, our study observed significant changes in the POSAS scores even one month after treatment.

When we compared the percentage change in the POSAS-O and POSAS-P scores regarding the scar site, we found that facial scars showed more improvement than those on the neck and hands, and this difference was statistically significant (p-value = 0.0007). Studies by Keen et al. [12] and El-Zawahry et al. [14] reported similar results.

A statistically significant relationship (p-value = 0.0120) was observed between the percentage change in the POSAS-O and POSAS-P scores in relation to the scar site among all three groups. The maximum improvement was seen in the post-traumatic group, followed by the post-burn and post-acne groups. Scars on other sites also showed improvement, but it was not statistically significant. Thus, FCL has better effects on post-traumatic scars than on post-burn or post-acne scars.

Regarding the age of scars, no statistical significance was found in scars older than five years. However, for scars less than five years old, a statistically significant difference (p-value = 0.0002) was observed in the patient scores, indicating that early scars (<5 years) showed substantial improvement in the patient part of POSAS, and that post-acne scars demonstrated statistically significant improvement over post-traumatic and post-burn scars during patient assessment.

The maximum improvement in observer scores was seen in middle-aged (6-10 years) scars, followed by younger (1-5 years) and older (>10 years) scars. In patient scores, the maximum improvement was also noted in middle-aged scars, followed by older and younger scars. Both patient and observer scores showed an insignificant relationship. Therefore, according to our study, the age of the scar does not significantly impact the outcome of laser treatment, as also evidenced in the study by Keen et al. [12].

Tolerance of the patients to the procedure was good, and there was minimal pain reported post-procedure. The most common complication, aside from pain, across all three groups was erythema, followed by crusting and edema. Although there was no significant difference among the three groups overall, the differences in the means of incidences of erythema and crusting were statistically significant (p-value = 0.0222). Therefore, following pain, erythema was the most commonly observed complication. Studies by Keen et al. [12], Majid et al. [13], and Issler-Fisher et al. [18] have reported similar findings.

At our center, three sessions of fractional CO_2_ laser therapy were conducted at four-week intervals for all patients, regardless of the etiology of the scars. The scars were located on various parts of the body. Three months after the last session, improvements were observed in skin texture, pigmentation, and overall appearance, with minimal and self-limiting side effects.

However, there were certain limitations in our study. We excluded hypertrophic scars, necessitating the exclusion of the parameters of thickness and surface area from the POSAS-O, each assigned a score of 1. Similarly, the parameter assessing the difference in thickness of the scar from normal skin in POSAS-P was excluded and assigned a score of 1. Additionally, our study had fewer participants than expected, the sex ratio was disproportionately higher for female patients, and the patient group was predominantly younger. Furthermore, fixed, predetermined laser parameters were used for all patients, which may have resulted in some being undertreated and others overtreated.

## Conclusions

Fractional CO_2_ laser therapy is an effective and swift treatment modality for scars, boasting a good safety profile with low long-term complications. It notably improves the quality of scars and produces significant enhancements in skin texture, showing better effects on post-traumatic scars compared to post-burn and post-acne scars. The procedures at our center were conducted without any form of anesthesia, indicating good pain tolerance among patients. The present study is among the few conducted in a resource-limited country in the medical field like India that assesses scars using the POSAS, which may contribute to further refinements of this scale. However, the results of our study need further validation in future research with proper randomization and a larger sample size. Additional studies are required to better understand the mechanism of action and to optimize the doses and timing of therapy.

## Figures and Tables

**Figure 4:**
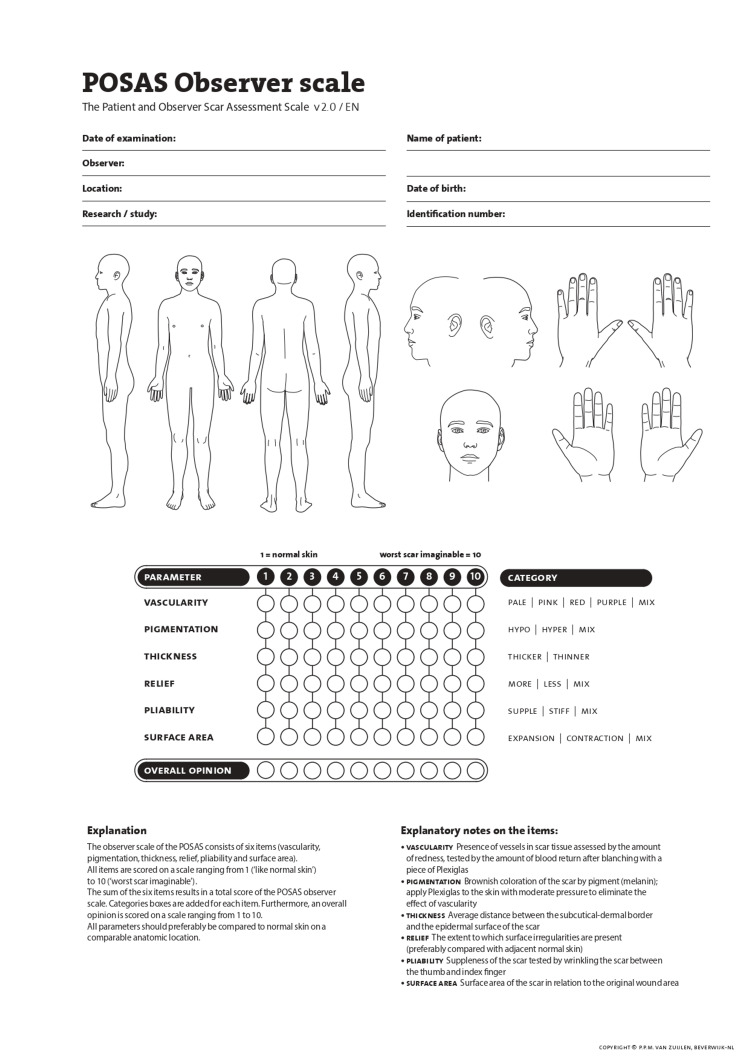
POSAS observer scale POSAS: Patient and Observer Scar Assessment Scale [19-20]. Permission to use the POSAS scale has been granted by the POSAS team, the Netherlands.

**Figure 5:**
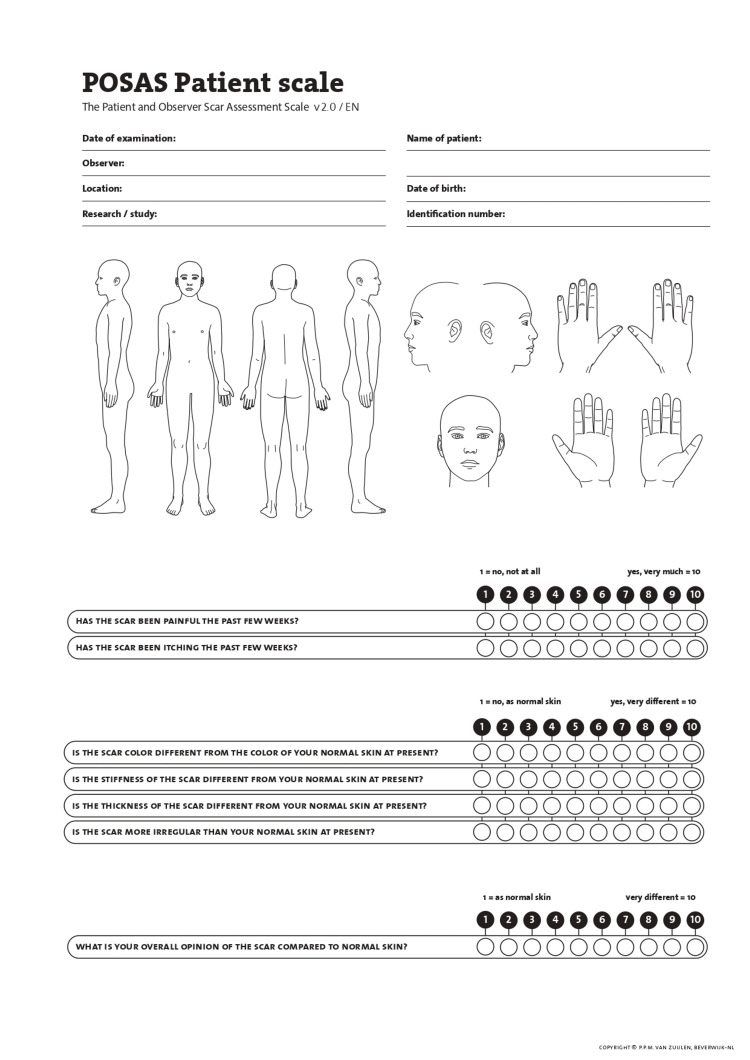
POSAS patient scale POSAS: Patient and Observer Scar Assessment Scale [19-20] Permission ro use the POSAS scale has been granted by the POSAS team, the Netherlands.
